# Dual Effect of 4-Methylumbelliferone on INS1E Cells: Enhancing Migration and Glucose-Stimulated Insulin Secretion

**DOI:** 10.3390/ijms26157637

**Published:** 2025-08-07

**Authors:** Giorgia Adamo, Daniele Romancino, Paola Gargano, Marta Sarullo, Aldo Nicosia, Sabrina Picciotto, Giulia Smeraldi, Antonella Bongiovanni, Monica Salamone

**Affiliations:** 1 Cell-Tech HUB, Institute for Research and Biomedical Innovation, National Research Council of Italy (CNR), Via Ugo La Malfa 153, 90146 Palermo, Italy; giorgia.adamo@irib.cnr.it (G.A.); daniele.romancino@irib.cnr.it (D.R.); paola.gargano@ibf.cnr.it (P.G.); marta.sarullo01@universitadipavia.it (M.S.); aldo.nicosia@irib.cnr.it (A.N.); sabrina.picciotto@ibf.cnr.it (S.P.); giulia.smeraldi@irib.cnr.it (G.S.); antonella.bongiovanni@irib.cnr.it (A.B.); 2 Department of Biological, Chemical and Pharmaceutical Sciences and Technologies (STEBICEF), University of Palermo, Viale delle Scienze, Ed. 16, 90128 Palermo, Italy

**Keywords:** cell adhesion, insulin secretion, 4-MU (4-methylumbelliferone), three-dimensional microenvironment (3D), type 1 diabetes

## Abstract

Recent studies have demonstrated that the coumarin derivative 4-Methylumbelliferone (4MU) has an antidiabetic effect in rodent models. 4MU is known to decrease the availability of hyaluronan (HA) substrates and inhibit the activity of different HA synthases. Nevertheless, it has been observed that 4MU may also affect cellular metabolism. In this study, we utilize the rat insulinoma beta cell line (INS-1E) cultured in both two-dimensional (2D) and three-dimensional (3D) experimental settings (pseudo islets), as an in vitro model to study beta cell functionality. For the first time, we observed that treating INS1E cells with 4MU results in improved insulin secretion. Additionally, we discovered that 4MU treatment elicited morphological changes from multilayer to monolayer conditions, along with a varied distribution of insulin granules and cell adhesion properties. Notably, we found that insulin secretion is not correlated with HA production. The same result was observed in co-culture experiments involving INS-1E cells and stromal vascular fraction (SVF) from adipose tissue. These experiments aim to investigate the effects of 4MU on beta cells in the context of its potential use in early-stage type 1 diabetes and in enhancing islet transplantation outcomes.

## 1. Introduction

Diabetes mellitus (DM) encompasses a group of chronic disorders characterized by impaired glucose metabolism, which is regulated by pancreatic beta cells [1]. The formation of spheroidal pseudo-islets combined with their co-culture with other cell types in three-dimensional (3D) systems using innovative biomaterials represents a promising strategy for studying and developing new technologies to improve the efficacy of cell-based therapies for type 1 diabetes [2,3,4,5,6,7]. It is also used as an in vitro model for screening antidiabetic substances and developing alternative therapies [8]. The functionality and long-term survival of pancreatic beta cells are intricately linked to their surrounding microenvironment and their interactions with the extracellular matrix (ECM). These interactions play a critical role in maintaining beta-cell homeostasis, supporting their ability to sense glucose levels and regulate insulin secretion effectively. Beta cells exhibit a well-defined cell polarity and possess specialized junctional complexes, including adherent junctions and integrin-mediated junctions, which are essential for maintaining tissue integrity and facilitating communication with adjacent cells [9]. Both cadherin-mediated and integrin-mediated cell junctions have been extensively studied and are known to contribute significantly to the regulation of insulin secretion. Cadherin-mediated adhesion supports beta-cell clustering and communication, whereas integrin-mediated signaling facilitates interactions with the ECM, influencing key cellular functions such as proliferation, survival, and insulin release [10]. Moreover, insulin granule secretion is a spatially regulated process that occurs at specific sites on the plasma membrane. These secretion events are localized within adhesion plaques that are highly enriched with integrin, reinforcing the importance of cell-ECM interactions in modulating insulin exocytosis. Understanding these complex regulatory mechanisms provides valuable insights into beta-cell biology and may contribute to the development of novel therapeutic strategies for diabetes treatment [11].

Modifying the ECM of islets has been demonstrated to significantly influence their function, affecting key cellular processes such as insulin secretion, survival, and communication with surrounding cells. Notably, in both type 1 and type 2 diabetes, inflammatory signals play a crucial role in driving disease progression by altering the islet microenvironment. Recently, 4MU has been identified as an inhibitor of HA synthesis in the in vivo model of diabetic mice and has demonstrated antidiabetic effects [12]. However, it remains unclear whether these effects are directly mediated by beta cells or by other cell types present in the islets of Langerhans. Within this context, research has shown that levels of HA are notably elevated in the serum and muscle tissues of individuals diagnosed with type 2 diabetes. However, this increase in HA levels is not observed in individuals with type 1 diabetes, suggesting differences in the ECM composition and inflammatory responses between the two forms of the disease. Moreover, during the development of type 1 diabetes, there is a substantial increase in HA around peri- and intra-islet micro vessels and in leukocytic infiltrates [13]. Recent studies have also revealed an interesting role of HA in islet engraftment after transplantation. Specifically, inhibiting HA using 4MU has been shown to have an inhibitory effect on post-transplant rejection of the islets of Langerhans, mediated by CD4 T cells [14]. Despite its seemingly simple structure, HA exerts numerous effects, including the regulation of cell growth, migration, and survival, as well as involvement in various pathological processes. The orchestration of these functions by HA remains partially unclear, although its molecular mass, turnover, and receptors are critical for its biological activities. HA is typically synthesized as a high-molecular-weight polymer (HMW HA). However, under pathological conditions such as cancer, inflammation, oxidative stress, and tissue remodeling, endogenous HMW HA can undergo accelerated degradation by hyaluronidases and reactive oxygen species [15]. Notably, UDP-sugars have been implicated in post-translational modifications [16]. Specifically, this study examines how 4MU treatment influences INS1E cell morphology and adhesiveness in both 2D and 3D culture conditions. Additionally, we explore the effects of 4MU on basal and glucose-stimulated insulin secretion by assessing its role in granule trafficking, a crucial process in regulated exocytosis. Beyond cell-intrinsic mechanisms, beta-cell function is also shaped by interactions with the surrounding stromal microenvironment. The stromal vascular fraction (SVF), which consists of various cell types including endothelial cells, immune cells, and fibroblasts, is a key player in ECM remodeling and HA production. To further elucidate the role of HA in beta-cell physiology, we investigated the co-culture of INS1E cells with SVF cells, evaluating how this interaction influences HA distribution and beta-cell function. Together, these studies contribute to a deeper understanding of the extracellular factors that regulate beta-cell behavior and may provide insights into potential therapeutic strategies targeting the ECM in diabetes.

## 2. Results

### 2.1. 4MU Treatment Influences Morphology and Adhesiveness in INS1E Cells Cultivated in 2D and 3D Conditions

**Figure 1:**
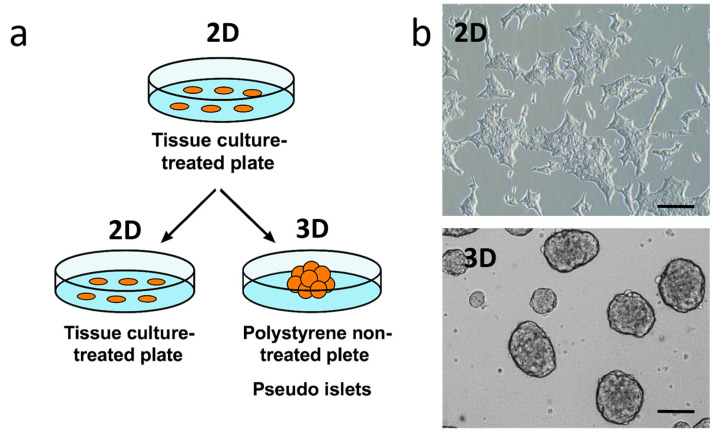
(**a**) Schematic representation of 2D and 3D pseudo-islet formation from INS1E cells. For two-dimensional (2D) culture, INS1E cells were seeded on an adherent plate (tissue culture-treated plate); for three-dimensional (3D) culture, INS1E cells were seeded non-adherent plate (polystyrene non-treated plate). (**b**) Phase contrast images of INS1E cells grown in 2D and 3D conditions after 4 days of incubation. Scale bar: 100 μm.

To investigate the impact of the ECM, including cell-cell and cell-matrix interactions, on the regulation of insulin secretion, we cultured INS1E cells under both 2D and 3D conditions. Under 2D culture conditions, INS1E cells grow forming aggregates with a multilayer morphology. When cultured on a non-adherent dish, they form 3D pseudo-islets ranging in size from 50 to 300 µm, resembling Langerhans islets (Figure 1).

**Figure 2:**
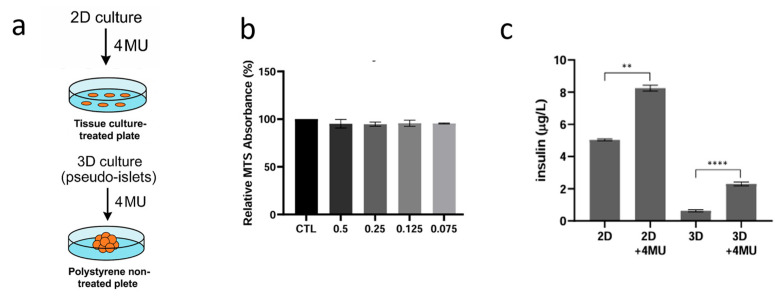
(**a**) Schematic representation of 2D and 3D INS1E cells treatment with 4MU. (**b**) Cell viability assessment (MTS assay) of INS1E cells treated with different concentrations of 4MU (0.5–0.075 mM) for 48 h. (**c**) Quantification of basal insulin secretion in INS1E cells grown in 2D and 3D conditions, with or without 0.250 mM of 4MU. Insulin levels were normalized to the total protein content (mg) of cell extracts. Data are presented as mean ± SD from three independent experiments. One-way ANOVA with Tukey’s multiple comparisons test was used to assess the statistical significance of differences between untreated and treated with 4MU, showing **** *p* < 0.0001 and ** *p* < 0.05. Scale bar: 100 μm.

Once 2D and 3D in vitro models were established, we investigated the direct impact of 4MU on INS1E cells. Initially, we show that treatment with various concentrations of 4MU (ranging from 0.075 to 0.5 mM) did not affect cell proliferation and viability. (Figure 2b). To further investigate the direct effects of 4-MU on pancreatic beta cells, particularly in relation to insulin secretion, INS1E cells were cultured under normoglycemic conditions (5.5 mM glucose) in both 2D and 3D systems for four days. After this period, the cells were treated with 4MU for 24 h, resulting in a clear increase in insulin secretion in both culture conditions (Figure 2c).

**Figure 3:**
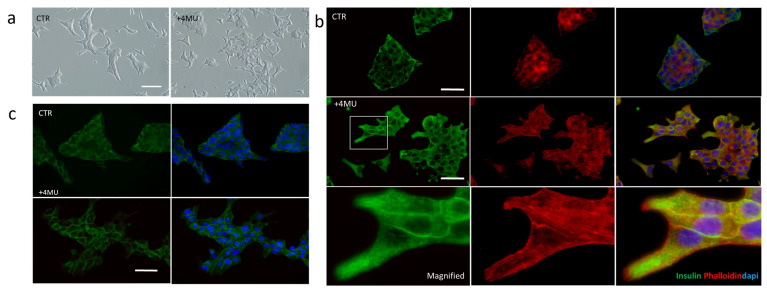
(**a**) Phase contrast images of INS1E cells grown in 2D conditions, with or without 0.250 mM 4MU cultivated on a tissue culture plate. Scale bar: 100 μm. (**b**) Immunofluorescence analysis of INS1E cells cultured in 2D conditions, with or without 4MU treatment, stained with anti-insulin antibody, DAPI, and Phalloidin. Scale bar: 50 μm. (**c**) Immunofluorescence staining for β-catenin and DAPI. Scale bar: 50 μm.

Notably, basal insulin secretion was higher in 2D cultures compared to 3D cultures. However, the stimulatory effect of 4MU was significantly more evident in the 3D environment, leading to a 1.6-fold increase in insulin secretion in 2D cultures and a remarkable 3.8-fold increase in 3D cultures. These findings suggest that while 2D cultures exhibit inherently higher baseline insulin secretion, 3D cultures demonstrate a heightened sensitivity to 4MU. In order to evaluate the effects of 4MU on cell morphology and adhesiveness, INS1E cells were observed after 48 h of treatment (Figure 3a), and 4MU-induced changes in cell morphology and behavior are detectable in two-dimensional in vitro culture systems. Specifically, cells treated with 4MU tend to grow as a monolayer with migratory morphology, with respect to the control cells that maintain a multilayered morphology. This suggests a change in cells adhesive properties of the cells. To further investigate the relationship between insulin secretion and changes in cell morphology, immunofluorescence analysis was performed on INS1E cells cultured in 2D, either treated or untreated with 4MU (Figure 3b).

Given that β-catenin, cadherins, and integrins have been reported to play a role in insulin granule exocytosis, we further explored the impact of 4MU on cell adhesion and junctional integrity. Immunofluorescence localization assays show that β-catenin, a key component of adherent junctions, is more localized at cell-cell contacts in 4MU-treated cells compared to control cells. (Figure 3b). Moreover, cells were also immunostained with an anti-insulin antibody (green signal) to visualize the localization of insulin granules (Figure 3c). Insulin granules are typically dispersed throughout the cytoplasm, with a subset positioned near the plasma membrane, ready for exocytosis (CTR, Figure 3c). INS1E cells treated with 4MU exhibited a more pronounced localization of insulin granules near the plasma membrane, particularly in proximity to membrane protrusions. This data suggests that 4MU treatment may enhance the mobilization of insulin granules towards the cell periphery, potentially facilitating their exocytosis. This observation was further supported by phalloidin staining (red fluorescence), which highlighted the presence of actin-rich protrusions specifically in 4MU-treated cells (Figure 3c). Notably, co-localization analysis revealed that insulin granules were closely associated with actin structures only in treated cells, whereas in untreated cells, insulin granules remained primarily distributed within the cytoplasm.

### 2.2. Study of 4MU Effects on Basal and Glucose-Stimulated Insulin Secretion (GSIS) Through Modulation of Granule Trafficking in INS1E Cells Cultured in 3D

**Figure 4:**
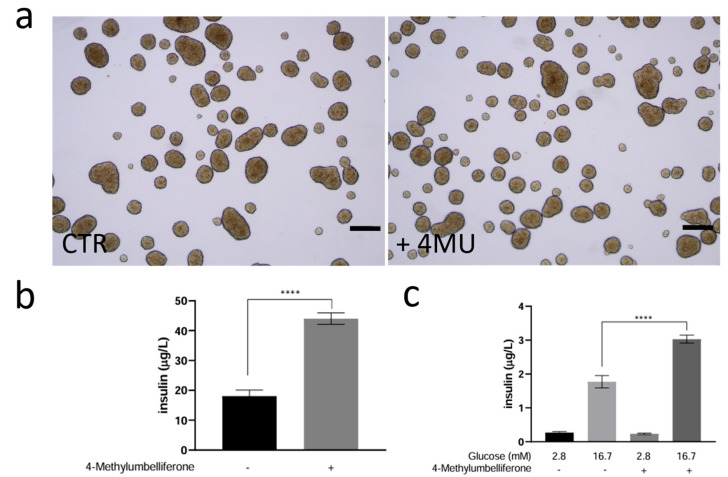
(**a**) Phase contrast Images of INS1E pseudo-islets treated or untreated with 4MU for 24 h. (**b**) Quantification of basal insulin secretion by INS1E cells cultured in 3D with or without 4MU after 24 h under normoglycemic conditions. (**c**) Quantification of insulin secretion from the same number of pseudo-islets after treatment with glucose at 2.8 mM for 60 min and 16.7 mM for 60 min. Values are expressed as the mean ± SD of three independent experiments. Unpaired *t* test (**b**) and One-way ANOVA (**c**) through Tukey multiple comparisons test, were used to assess the statistical significance of differences between untreated and treated with 4MU, showing **** *p* < 0.0001 for all conditions. Scale bar: 200 μm.

To investigate the potential impact of 4MU treatment on insulin granule secretion following glucose stimulation, we analyzed the kinetics of glucose-induced insulin release in 4MU-treated INS1E 3D cultured (pseudo-islets). Pseudo-islets were produced as described in the Materials and Methods, and after 5 days in culture, were treated with 4MU for 24 h under normoglycemic conditions. We did not observe any evident morphological differences (Figure 4a); however, a significant increase in insulin release was detected after treatment (Figure 4b). Then, we followed a well-established approach to assess glucose-stimulated insulin secretion (GSIS). In this method, basal glucose (2.8 mM) mimics fasting conditions, while high glucose (16.7 mM) is used to evaluate the β-cell response to increased glucose availability.

As shown in Figure 4c, both untreated and 4MU-treated pseudo-islets demonstrate an increased ability to release insulin in response to high glucose concentrations; however, this increase is significantly higher in 4MU-treated pseudo-islets. Additionally, the Stimulation Index (SI ≠ insulin released at high glucose/insulin released at low glucose) is significantly higher in the 4MU-treated pseudo-islets (SI = 14.2 ± 1.37) than in the control (SI = 7.62 ± 1.76), indicating improved glucose sensitivity. The results suggest that 4MU treatment enhances the pseudo-islets’ responsiveness to high glucose levels, leading to greater insulin secretion compared to the control.

### 2.3. Co-Culture of INS1E with Stromal Vascular Fraction and the Role of HA

**Figure 5:**
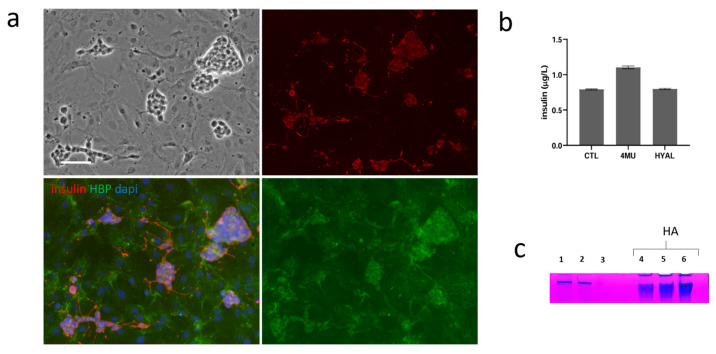
(**a**) Immunofluorescence analysis of the INS1E/SVF co-culture in 2D conditions after 7 days, with staining for DAPI (nuclei), insulin, and Hyaluronic binding protein (HBP). Scale bar: 200 μm. (**b**) Quantification of basal insulin secretion in INS1E/SVF co-cultures treated or not with 4MU and hyaluronidase (6 U/mL). (**c**) Electrophoresis analysis and staining of HA extracted from conditioned medium by the same cells, in the presence or absence of 4MU and HYAL (6 U/mL). The analyzed samples include: (1) INS1E/SVF control, (2) INS1E/SVF treated with 4MU, (3) INS1E/SVF treated with HYAL (6 U/mL). As a control, commercial HA was loaded at increasing concentrations in µg: 1 (4), 1.5 (5), 2 (6).

We used a co-culture of stromal vascular fraction (SVF) cells from adipose tissue and INS1E cells as an in vitro model to mimic the complex cellular interactions within the islet transplantation microenvironment. Using this system, we evaluated the effects of 4MU on insulin secretion and HA production. An equal number of INS1E and SVF cells were seeded in 24-well plates and cultured for seven days. As shown in Figure 5a, INS1E cells were stained with an anti-insulin antibody to detect insulin within the co-culture, while hyaluronic acid binding protein (HBP) staining was used to visualize HA distribution. HBP staining confirmed the presence of HA in both INS1E and SVF cells, confirming that both cell types contribute to its production or interaction. In contrast, insulin was exclusively localized in INS1E cells, particularly in membrane protrusions. This indicates that while both cell types contribute to shaping the extracellular environment, including HA dynamics, insulin secretion remains a unique function of beta cells.

To assess whether 4MU treatment could reduce HA content, we also treated INS1E/SVF co-cultures with hyaluronidase (HYAL), an enzyme that digests HA, as a control. Notably, 4MU treatment led to a significant increase in insulin secretion, an effect not observed in HYAL-treated cells (Figure 5b). We further analyzed the conditioned medium from INS1E/SVF co-cultures to evaluate differences in HA release under different treatment conditions. As shown in Figure 5c, SDS-PAGE gels stained with a specific HA dye revealed a single band corresponding to the molecular weight of a commercial HA standard at various concentrations (bands 4, 5, and 6, Figure 5c). The band profiles of control and 4MU-treated INS1E/SVF cultures were comparable (bands 1 and 2, Figure 5c), while the band was completely absent in HYAL-treated cells (band 3, Figure 5c), indicating complete HA degradation.

## 3. Discussion

INS1E cells replicate several key aspects of primary beta cells by forming pseudo-islets under 3D conditions. Notably, their oxygen consumption rates and mitochondrial redox responses to glucose and metabolic modulators closely resemble those of pancreatic islets [17]. This model enables the study of dynamic insulin secretion patterns comparable to those observed in primary rat islets [18]. Moreover, the limited availability of high-quality human pancreatic islets for research purposes, along with variations in their relative cell type composition, makes this system useful for in vitro study. In our study, we observed that treatment with 4MU induces changes in the adhesiveness of INS1E beta cells, as well as an increase in insulin secretion. In cells treated with 4MU, insulin predominantly accumulates at regions where cells establish direct contact with each other and at sites of interaction between the cells and the ECM. This observation underscores the critical role that cellular interactions play in the regulation of insulin secretion. The localization of insulin within these specific regions suggests that both cell-cell adhesion and cell-matrix signaling contribute to the proper trafficking and release of insulin granules. Previous research has indicated that structural abnormalities in the ECM, as well as disruptions in cell-cell junctions, may have profound effects on cellular function, potentially leading to various pathological conditions [19]. These alterations are thought to interfere with the organization of the actin cytoskeleton, a key component in maintaining cell shape, adhesion, and intracellular transport processes. In the highly organized three-dimensional structure of pancreatic islets in vivo, proper cell-cell interactions and actin cytoskeletal rearrangement are believed to be essential for the formation and maintenance of an insulin granule pool that is responsive to glucose stimulation. The coordination of these structural elements ensures the efficient mobilization and release of insulin in response to changes in blood glucose levels. Therefore, disruptions in these cellular components could contribute to beta-cell dysfunction and impaired insulin secretion, which are key features of diabetes pathophysiology [20]. In our study, we observed that following treatment with 4MU, beta-catenin is more prominently localized at cell-cell contact points. This observation is particularly interesting because recent research has shown that adherent junctions mediated by beta-catenin regulate a reservoir of insulin-containing secretory vesicles [21,22,23]. Our findings suggest that 4MU treatment not only enhances insulin secretion but also alters the cellular architecture, potentially by reorganizing the actin cytoskeleton and modulating adhesion molecules involved in granule trafficking. Moreover, the pronounced effects observed in a three-dimensional environment may be attributed to enhanced cell-cell interactions and a more physiologically relevant cellular organization, further supporting the role of 4MU in modulating insulin secretion dynamics. This enhanced response in the 3D system may be attributed to its more physiologically relevant microenvironment, which better mimics in vivo conditions and thus fosters greater responsiveness to external modulators like 4-MU. Thus, the increased insulin levels observed in our study are most likely attributable to enhanced granule mobilization and exocytosis, rather than increased de novo insulin biosynthesis [21]. However, to conclusively delineate the relative contributions of these processes, further investigations will be necessary. In particular, pathway-focused gene expression analyses, targeting key regulators of β-cell identity, insulin biosynthesis, vesicle trafficking, and exocytosis, will be instrumental in clarifying the molecular mechanisms underlying the effects of 4MU. Such studies, potentially involving transcriptomic profiling or targeted qRT-PCR panels, may also help identify specific signaling cascades modulated by 4MU and provide a more comprehensive view of its impact on β-cell function and phenotype. In this context, it is worth noting that efforts to preserve or restore β-cell function are also at the core of advanced therapeutic strategies, such as islet cell transplantation. Islet cell transplantation is regarded as a promising treatment for insulin-deficient diabetes. Mesenchymal stem/stromal cells have been shown to secrete different paracrine molecules with antiapoptotic properties that act by improving, in a co-culture system, the viability and function of the Langerhans islets. The co-culture of stromal vascular fraction (SVF) cells from adipose tissue and pancreatic beta cells represents a promising model for investigating beta-cell protection, regeneration, and functional improvement [7]. To further investigate the effect of 4MU, we co-cultured SVF cells derived from adipose tissue with INS1E cells. Our findings reveal that, in co-culture conditions and following a treatment with 4-MU and HYAL, increased insulin secretion is related to 4-MU treatment, since this change is absent in samples treated with HYAL. Additionally, our observations suggest that in this system, the effects of 4-MU are not directly linked to HA production but to other metabolic processes. Specifically, the impact of 4-MU might be attributed to the accumulation of its by-products and their subsequent involvement in alternative metabolic pathways [24]. A suggested major mechanism for 4-MU to inhibit HA production is via its function as a competitive substrate for UDP-glucuronosyltransferase to synthesize UDP-N-acetyl glucosamine (UDP-GlcNAc), one of the two substrates for HA synthesis [25]. Moreover, UDP-GlcNAc is also extensively involved in intracellular signalling as a substrate for O-linked N-acetyl glucosamine transferases, and it serves as a substrate for making glycosaminoglycans, proteoglycans, and glycolipids as well. Moreover, 4-MU reduces intracellular levels of UDP-glucuronic acid, which plays a key role in regulating sugar metabolism. This depletion can disrupt the balance of activated sugars and modify the metabolic flux of the glycolysis pathway. Since glycolysis serves as a primary pathway for ATP production, the treatment with 4-MU may lead to an increase in ATP generation, which is a crucial signaling molecule for insulin release in pancreatic beta cells. The elevated levels of ATP can enhance various cellular processes, including those related to adhesion dynamics and insulin secretion. Given that ATP availability directly influences the activity of focal adhesion-associated proteins, it is possible that 4-MU treatment promotes the activation of key regulators such as Focal Adhesion Kinase (FAK) and paxillin [26]. These proteins play an essential role in modulating focal adhesion turnover and intracellular signaling pathways that govern insulin granule trafficking and secretion. Moreover, an increase in focal adhesion stability may lead to stronger anchoring of beta cells to the ECM, thereby optimizing their structural support and functional capacity for insulin release. More stable adhesion sites could facilitate efficient vesicle docking and exocytosis, ultimately enhancing the responsiveness of beta cells to glucose stimulation. In addition to cell-matrix interactions, intercellular contacts among beta cells are equally important for synchronized insulin secretion. Strengthened cell-cell junctions, mediated by adhesion molecules such as cadherins and integrins, may improve the coordination of insulin release across the beta-cell population. This ensures a more uniform and effective response to glucose fluctuations, supporting overall islet function and glucose homeostasis. While additional studies are certainly required to elucidate the precise molecular mechanisms by which 4MU acts on β-cells, particularly in primary rodent and human islets, our current findings provide compelling preliminary evidence supporting its potential therapeutic role. Taken together, our data highlight the translational relevance of 4MU and lay the groundwork for future investigations aimed at defining its mechanisms of action and optimizing its clinical application.

## 4. Materials and Methods

### 4.1. Cell Culture

The rat insulinoma beta cell line (INS-1E) [27], was cultured in a humidified atmosphere containing 5% CO_2_ and at 37 °C in a complete medium, composed of RPMI-1640 medium (Sigma-Aldrich, Saint Louis, MO, USA, R8758) containing 10% FBS (Sigma-Aldrich, ES-009-B), 2 mM L-Glutamine (Sigma-Aldrich G7513), 10 mM HEPES (Sigma-Aldrich, TMS-003-C), 1 mM Sodium Pyruvate (Sigma-Aldrich, TMS-005-C), 50 µM 2-Mercaptoethanol (Sigma-Aldrich, M6250), penicillin 100 U/mL (Sigma-Aldrich) and streptomycin (Sigma-Aldrich). In order to compare 2D INS1E cells and 3D INS1E pseudo-islets, 1 × 10^6^ INS1E were seeded on a tissue culture-treated plate (Corning CLS430167, Corning, NY, USA) for two-dimensional (2D) culture INS1E cells; and a polystyrene non-treated plate (Corning CLS430591) for three-dimensional (3D) culture INS1E cells were seeded.

### 4.2. Isolation of Stromal Vascular Fraction from Rat Adipose Tissue

Tissue Wistar female rats (average body weight of 350 g) were used for the stromal vascular fraction. The epididymal adipose tissue was minced in small pieces and mixed with a solution of Dulbecco’s Modified Eagle Medium (DMEM, Sigma-Aldrich, Milan, Italy) without serum, to which were added 150 U/g Collagenase H (Abiel, Palermo, Italy) and 10 μg/mL Thermolysin (Promega, Madison, WI, USA) (10 mL/g of tissue). Samples were incubated at 37 °C for 2 h in static conditions. After the incubation time, samples were centrifuged to remove adipocytes, and the pellet containing the vascular fraction was resuspended in 30 mL of DMEM with 10% (*v*/*v*) Fetal Bovine Serum (Euroclone, Milan, Italy) to stop the collagenase activity. The solution was then centrifuged at 300× *g*, 10 min at r.t. After discarding the supernatant, the pellet was resumed in 30 mL of DMEM 10% FBS and centrifuged at 300× *g*, 10 min at r.t. This step was repeated 3 times to wash the cells, and in the end, the pellet was resumed in 10 mL DMEM with 10% FBS. The cells were used starting from passage 3.

### 4.3. Quantification of Basal Insulin

In order to quantify the basal insulin, both in 2D and 3D, the same number of INS1E cells (1 × 10^6^ cells) were seeded on a tissue culture-treated plate (Corning CLS430167) for two-dimensional (2D) culture; and on a polystyrene non-treated plate (Corning CLS430591) for three-dimensional (3D). After 4 days in culture, cells and pseudo-islets were treated 24 with or without 4-MU (HYN0187-MedChemExpress, Monmouth Junction, NJ, USA) and HYAL (S-HX0514-1 Sigma-Aldrich). Each conditioned medium was collected and stored at −20 °C. The cells were then extracted with Triton 0.5% containing protease inhibitors for 30 min on ice and then centrifuged at 10,000 rpm for 10 min and stored at −20 °C. The insulin secretion was quantified by Rat ELISA kit (Mercodia, Uppsala, Sweden, 10-1250-01) according to the manufacturer’s instructions. The conditioned medium has been diluted 50 times in order to be quantified. In order to normalize the insulin release/mg protein, we quantify protein from cell extracts using the microBCA protein assay kit (Thermo Fisher Scientific, 23225, Waltham, MA USA) according to the manufacturer’s instructions, using bovine serum albumin (BSA) as a standard. Then, the samples and standards were mixed with the working reagent and incubated for 1 h at 37 °C. Absorbance values were measured at 562 nm, using a GloMax^®^ Discover Microplate Reader (Promega Corporation, Madison, WI, USA).

### 4.4. Glucose Stimulated Insulin Secretion (GSIS) Assay

INS1E cells were plated at 1 × 10^6^ cells on a polystyrene non-treated plate (100 mm × 20 mm) to obtain pseudo-islets. After four days in culture, pseudo-islet was treated with 4MU 0.250 mM for 24 h.; pseudo-islet treated and not treated were washed with PBS and pre-incubated for 30 min at 37 °C in glucose-free Krebs buffer supplemented with 0.5% BSA and containing (in mM) 119 NaCl, 4.74 KCl, 1.19 MgCl_2_, 2.54 CaCl_2_, and 25 HEPES. The glucose-free buffer was then replaced with the same buffer supplemented with either 2.8 or 16.7 mM glucose, and cells were incubated for 60 min at 37 °C. Supernatants containing released insulin were collected and stored at −20 °C until analysis, which was performed using a rat ELISA Kit (Mercodia, Uppsala, Sweden, 10-1250-01) by following the manufacturer’s instructions. The insulin quantification was made without dilution.

### 4.5. Cell Viability

Cell viability was measured by MTS assay (Promega, PR-G3582). MTS [3-(4,5- dimethylthiazol-2-yl)-5-(3carboxymethoxyphenyl)-2-(4-sulphophenyl)- 2H-tetrazolium] was utilized according to the manufacturer’s instructions. Cells were seeded at a density of 2 × 10^4^ cells/well on 96-well plates. After the treatment with different doses of 4MU for 48 h, 20 μL of the MTS solution was added to each well, and incubated for 4 h at 37 °C, 5% CO_2_. As a control, we used DMSO. The absorbance was read at 490 nm using a GloMax^®^ Discover Microplate Reader.

### 4.6. Immunofluorescence Analyses

INS1E cells were cultured in the different conditions and fixed in 3.7% formaldehyde (Sigma-Aldrich) for 10 min and permeabilized with 0.1% Triton X-100 (Sigma-Aldrich) for 5 min. The target proteins were recognized by the following monoclonal primary antibodies: anti-beta-catenin (1:300), anti-insulin (SAB4200691 Sigma-Aldrich). αActin was detected using Alexa fluor phalloidin. The cell nuclei were stained by DAPI (Sigma-Aldrich). The HA was stained with Hyaluronic Acid Binding Protein (S-385911-50UG Sigma-Aldrich), and ExtrAvidin (R)-FITC (S-E2761-.2ML, Sigma-Aldrich). Cells in 2D and 3D conditions were, respectively, observed under an OLYMPUS BX50 Fluorescence microscope (Tokyo, Japan).

### 4.7. Quantification of HA

An equal number of INS1E and SVF cells (20,000 for each) were seeded on 24-well plates and cultured for seven days. After that, cells were exposed to 4-MU or HYAL for 24 h. Supernatants from INS1E cells (control and treated groups) were digested with proteinase K (100 μg/mL in the medium) at 60 °C for 60 min. To precipitate hyaluronan, three volumes of cold ethanol were added to each sample, which were then stored at −20 °C for 24 h. Following incubation, samples were centrifuged at 13,000× *g* for 20 min at 4 °C. The resulting pellets were resuspended in distilled water and analyzed by 10% sodium dodecyl sulfate-polyacrylamide gel electrophoresis (SDS-PAGE). Conditioned media samples, along with 3, 5, and 10 μg of standard HA (from Contipro, Dolní Dobrouč, Czech Republic), were prepared by mixing 10 μg of sample (in PBS) with the appropriate volume of 5× loading buffer (0.25 M Tris-Cl pH 6.8, 10% SDS, 50% glycerol, 0.25 M dithiothreitol (DTT), 0.25% bromophenol blue). Samples were then heated at 100 °C for 5 min and loaded onto a 10% SDS-PAGE gel. After electrophoresis, the gel was stained overnight with Stains-All (Sigma-Aldrich) prepared at a concentration of 20 mg/100 mL in 50% ethanol/water.

### 4.8. Statistical Analysis

GraphPad Prism version 8 was used for statistical analysis. *t*-test, one-way analysis of variance (ANOVA). Statistical significance was set as *p* < 0.05, and star significance was distributed as * for *p* < 0.05, ** for *p* < 0.01, *** for *p* < 0.001, and **** for *p* < 0.0001. Each measurement reported here was repeated as an experimental triplicate, and mean values, as well as standard deviations, were calculated.

## 5. Conclusions

Our study shows that treatment of INS1E beta cells with 4-MU induces significant changes in their adhesiveness, associated with a significant increase in insulin secretion. In INS1E-treated samples, insulin primarily localizes at cell-cell and cell-matrix contact points, emphasizing the importance of cellular interactions in regulating insulin secretion. We observed that 4-MU treatment leads to an increased localization of beta-catenin at cell-cell contacts, confirming the critical role of adherent junctions in regulating insulin secretory granules. These effects suggest the potential role of 4MU in modulating pancreatic β-cell function and suggest new possibilities for its use in regulating insulin secretion. Finally, our study underscores the necessity of further exploring the role of 4MU and its metabolic regulators in pancreatic islet transplantation and diabetes treatments. Enhancing cellular adhesion and secretory function could represent a significant step forward toward more effective and sustainable therapeutic strategies.

## Data Availability

The original contributions presented in this study are included in the article. Further inquiries can be directed to the corresponding authors.
